# The roles and regulatory mechanisms of TGF-β and BMP signaling in bone and cartilage development, homeostasis and disease

**DOI:** 10.1038/s41422-023-00918-9

**Published:** 2024-01-24

**Authors:** Mengrui Wu, Shali Wu, Wei Chen, Yi-Ping Li

**Affiliations:** 1 Department of Cell and Developmental Biology, College of Life Sciences, Zhejiang University, Hangzhou, Zhejiang China; 2 Division in Cellular and Molecular Medicine, Department of Pathology and Laboratory Medicine, Tulane University School of Medicine, Tulane University, New Orleans, LA USA

**Keywords:** Cell biology, Developmental biology

## Abstract

Transforming growth factor-βs (TGF-βs) and bone morphometric proteins (BMPs) belong to the TGF-β superfamily and perform essential functions during osteoblast and chondrocyte lineage commitment and differentiation, skeletal development, and homeostasis. TGF-βs and BMPs transduce signals through SMAD-dependent and -independent pathways; specifically, they recruit different receptor heterotetramers and R-Smad complexes, resulting in unique biological readouts. BMPs promote osteogenesis, osteoclastogenesis, and chondrogenesis at all differentiation stages, while TGF-βs play different roles in a stage-dependent manner. BMPs and TGF-β have opposite functions in articular cartilage homeostasis. Moreover, TGF-β has a specific role in maintaining the osteocyte network. The precise activation of BMP and TGF-β signaling requires regulatory machinery at multiple levels, including latency control in the matrix, extracellular antagonists, ubiquitination and phosphorylation in the cytoplasm, nucleus-cytoplasm transportation, and transcriptional co-regulation in the nuclei. This review weaves the background information with the latest advances in the signaling facilitated by TGF-βs and BMPs, and the advanced understanding of their diverse physiological functions and regulations. This review also summarizes the human diseases and mouse models associated with disordered TGF-β and BMP signaling. A more precise understanding of the BMP and TGF-β signaling could facilitate the development of bona fide clinical applications in treating bone and cartilage disorders.

## Introduction

Transforming growth factor-βs (TGF-βs) and bone morphometric proteins (BMPs) are cytokines belonging to the TGF-β superfamily. Around the 1970s, TGF-β was discovered as a growth factor (GF) that can transform mammalian fibroblasts.^1^ At the same time, BMP was found to be capable of inducing ectopic bone formation.^2^ TGF-β and BMP signaling regulates a variety of physiological and pathological processes. TGF-β and BMP signaling is also critical for skeletal system development and homeostasis, which has been comprehensively investigated by using cell and animal models and clinical studies. Numerous mutations of the genes in TGF-β and BMP signaling are associated with human skeletal disorders. Many mouse models with dysregulated TGF-β and BMP signaling displayed certain skeleton defects. In this review paper, we summarize the genetic mouse models (Table 1) and human diseases (Table 2) related to TGF-β and BMP signaling in the skeleton. We also comprehensively review the essential roles and dynamic regulatory functionality of TGF-β and BMP signaling in the skeletal system during embryonic development and postnatal homeostasis, mostly focusing on chondrocytes, osteoblasts, osteocytes, and osteoclasts.

**Table 1:** Mouse models of TGF-β and BMP signaling in bone.

Classification	Gene	KO/CKO/Tg/knock-in	Phenotype	References
TGF-β ligands	*Tgfb1*	KO	Early death (1 month)	^88^
		*Tgfb1*^*−/−*^*Rag2*^*−/−*^	Reduced bone density; OB↓	^152^
		Col1α1 Prom-Tgfb1 H222D Tg ^(1)^	Diaphyseal thickening, fluctuating bone volume, increased bone remodeling, prone to fracture; OB↑; OC↑	^152^
		Col1α1 Prom-Tgfb1 H222D Tg	Knee and temporomandibular joint osteoarthritis↑	^221,222^
	*Tgfb2*	KO	Perinatal mortality; neural arch defect; bifurcated sternum; shortened radius and ulna	^85^
	*Tgfb3*	KO	Die within 20 h of birth; failure of the palatal shelves to fuse leading to cleft palate	^86,87^
BMP ligands	*Bmp2*	Col2α1-CreER	Severe chondrodysplasia; shortened stature and limbs; chondrocyte proliferation↓ & hypertrophy↓	^50^
		Prx1-Cre	Normal limb patterning	^51^
	*Bmp7*	KO	Die at birth; skull base defects; rib & sternum malformation; hindlimb polydactyly	^326^
	*Bmp7, Alk6*	DKO	Malformed and shortened appendicular bones compared to *Bmp7*^*−/−*^	^25^
	*Bmp2, 7*	*Prx1-Cre;Bmp2*^*f/f*^*;Bmp7*^*−/−*^	Slightly diminished appendicular skeleton; missing the last phalanx in digit III; malformed fibulae	^51^
	*Bmp4*	Col2α1-CreER	Mild chondrodysplasia	^50^
		Prx1-Cre	Polydactyly	^51,52^
	*Bmp2, 4*	Col2α1-CreER	Severe chondrodysplasia; severely shortened and malformed or missing long bone skeleton element & fused joints; chondrocyte proliferation↓ & hypertrophy↓	^50^
		Prx1-Cre	Polydactyly; complete syndactyly; delayed mineralization; chondrogenesis ↓ ; osteogenesis↓	^51^
	*BMP3/GDF10*	Col1 Prom-Bmp3 Tg	Late hypertrophic chondrocyte differentiation↓; thinner cortical bone; mineralization ↓ ; rib fracture	^115^
		KO	Increased bone density	^28^
	*Bmp14/GDF5*	bp^J (2)^	Normal axial bones; shortened limbs and digits; missing joints of autopods; missing phalange elements	^25,327^
			No delay in fracture healing	^328^
			Increased joint damage in collagen-induced arthritis; reduced bone density	^329^
	*GDF5, Alk6*	*bp*^*J*^*/bp*^*J*^*;Alk6*^*−/−*^	Same as bp^J^	^25^
Type I receptors	*Alk2/Acvr1*	Col2α1-Cre	Shortened cranial base; hypoplastic cervical vertebrae	^49^
		Osx-Cre	Mandibular bone density ↓ ; OB ↓ ; sRANKL ↑ ; OC↑	^113^
		Q207D Tg	Fibrodysplasia ossificans progressive	^127,130,132^
		*Acvr1*^*tnR206H/+*^;Tie2-Cre	Fibrodysplasia ossificans progressive	^128^
		R206H knock-in	Fibrodysplasia ossificans progressive	^129^
	*Alk3/Bmpr1A*	Col2α1-Cre	Split dorsal arches; shortened limbs; hypoplastic scapula; chondrodysplasia, chondrocyte proliferation ↓ , hypertrophy ↓ , terminal differentiation ↓ , and apoptosis↑	^48,49,65^
		Gdf5-Cre	Automatically develop osteoarthritis	^330^
		Col1α1-CreER	bone mass in long bones and ribs ↑ ; strength ↑ ; OC↓	^148,149^
		Sp7-Cre	Trabecular bone mass↑	^147^
		Dmp-Cre	Trabecular bone mass↑ (13-fold); OB proliferation↑ and activity ↓ ; RANKL & SOST ↓ ; OC↓	^147,176^
	*Alk6/Bmpr1B*	KO	Brachypodism; reduced phalangeal elements; the fusion of appendicular joints, similar to GDF5 mutant (bp^J^) mice	^25,48,49,65^
		KO	Transient and gender-specific osteopenia caused by reduced osteogenesis from MSCs	^331^
		Col1α1 Prom-truncated Alk6 Tg	Reduced BMD and bone volume; reduced osteoblast and osteoclast number	^332^
	*Alk3, Alk6*	*Col2α1-Cre;Alk3*^*f/f*^*;Alk6*^*+/−*^	Phenotype resembling and more severe than *Alk3* CKO mice	^48,65^
		*Col2-Cre;Alk3*^*f/f*^*;Alk6*^*−/−*^	Severe defects in cartilage formation and skeletogenesis	^48^
	*Alk2, Alk3*	Col2-Cre	Malformed axis skeleton (vertebra, cervical and thoracic regions); more severe appendicular defects than *Alk3* CKO mice	^49^
	*Alk2, Alk6*	*Col2-Cre;Alk2*^*f/f*^*;Alk6*^*−/−*^	More severe axis and appendicular defects than each single KO	^49^
	*Alk5/TGFBR1*	Dermo-Cre	Short and wide long bones, ectopic cartilaginous protrusions, reduced bone volumes	^83^
		Col2α1 Prom-Alk5 DN Tg	Elongated limbs; chondrocyte proliferation↑	^68^
		Col2α1-CreERT	Automatic osteoarthritis	^208,333^
Type II receptors	*Tgfbr2*	Nestin-CreER	Knee osteoarthritis↓	^221^
		ColX-Cre	Delayed chondrocyte terminal differentiation; impeded mineralization	^84^
		Prx-Cre	Die at birth; reduced periodontal and frontal bone; shortened limbs; split sternum; autopod joint fusion; reduced mineralization; chondrocyte proliferation↓ & hypertrophy↑	^78,81^
		Col2α1-Cre	Survive; neural arch defect; missing/incomplete intervetebral discs; progressive reduction in long bone length	^79,80^
		Sp7-Cre (Dox)	Early death (1 M); reduced body size; reduced bone volume; increased bone marrow adipose tissue; disrupted molar tooth formation; OB↓	^157,334^
		Ocn-Cre	bone density ↑ ; OB ↑ ; OC↑	^172^
	*Bmpr2*	Prx1-Cre	Trabecular bone volume ↑ ; BFR ↑ ; selectively abolish Activin-Smad2/3 but not Bmp-Smad1/5/8 signaling	^335^
		Col1α1 Prom-Bmpr2 DN Tg	Dwarfism; delayed mineralization; bone volume ↓ ; no change in cortical bone	^114^
	*ActRIIA/Acvr2A*	Ocn-Cre	Trabecular bone volume↑	^336^
	*ActRIIB/Acvr2B*	KO	Late hypertrophic chondrocyte differentiation ↓ ; thinner cortical bone; mineralization ↓ ; rib fracture	^115^
		Ocn-Cre	Normal	^336^
	*ActRIIA, ActRIIB*	Ocn-Cre	Trabecular bone volume↑ like *ActRIIA*^*−/−*^	^336^
Canonical pathway	*Smad1*	Col1α1-Cre	Osteopenia; OB proliferation and differentiation↓	^337^
		Col2α1-Cre	Shortened growth plate; chondrocyte hypertrophy↓ and proliferation↓	^68^
	*Smad1/5*	*Col2α1-Cre;Smad1*^*f/f*^*;Smad5*^*+/−*^	Similar and more severe phenotype than Smad1 CKO	^68^
	*Smad1/5*	Col2α1-Cre	Chondrodysplasia; shortened limbs; thicker perichondrium; matrix production ↓ ; hypertrophy↓	^69^
	*Smad8*	KO	Normal	^69^
	*Smad1/5/8*	*Col2α1-Cre;Smad1*^*f/f*^*;Smad5*^*f/f*^*;Smad8*^*−/−*^	Absence of an axial skeleton; severely disorganized appendicular bones	^69^
	*Smad2*	Col2α1-Cre	Similar and more severe phenotype than *Smad3* KO mice	^89^
	*Smad2/3*	*Col2α1-Cre;Smad2*^*f/f*^*;Smad3*^*−/−*^	Similar and more severe phenotype than *Smad3* KO mice	^89^
	*Smad3*	KO	Postnatal dwarfism; expanded columnar and hypertrophic zone; chondrocyte proliferation↑ and hypertrophy↑	^89^
		KO	Knee and temporomandibular joint osteoarthritis	^214,215^
		KO	Osteopenia; OB and OCY apoptosis↑	^156^
	*Smad4*	Tbx18-Cre	Short limbs, chondrogenesis↓ & hypertrophy ↓ , missing stylopod	^40^
		Sp7-Cre (Dox)	Increased trabecular bone mass	^147^
		Sp7-Cre	Stunted growth; spontaneous fractures; increased trabecular bone volume; decreased BMD; a combination of features seen in osteogenesis imperfecta, cleidocranial dysplasia, and Wnt-deficiency syndromes	^118^
		Dmp-Cre	Increased trabecular bone mass (~2-fold)	^147^
		Col1α1-Cre	Increased trabecular bone mass; protection from tail suspension-induced bone loss; OB & OCY number ↑ ; OB & OCY apoptosis↓	^178^
		Ocn-Cre	Lower bone mass < 6-month, more bone mass > 7-month	^177^
		Ctsk-Cre	Reduced bone mass; OC↑	^186^
Non-canonical pathway	*TAK1*	Col2α1-CreER	Growth retardation; osteoarthritis markers ↑ ; no osteoarthritis histological signs	^71^
		Osx-Cre	Cleidocranial dysplasia (CCD)-like phenotype (clavicular hypoplasia and delayed fontanelle fusion); OB ↓ ; reduced cancellous and trabecular bone volume	^116^
		Col2α1-Cre	Shorter limbs; chondrocyte proliferation ↓ , survival↓ & hypertrophy ↓ ; failure to maintain interzone cells of the elbow joint	^338^
		Prx1-Cre	Widespread joint fusions; chondrocyte hypertrophy and proliferation	^338^
	*p38*	Ocn-Cre	OB activity and BFR ↓ ; reduced cancellous and trabecular bone volume	^117^
		Col2α1 Prom-p38DN Tg	Shortened limbs; knee joint osteoarthritis↑	^210^
		*p38b*^*−/−*^	A substantial decrease in long bone mineralization and a more modest effect on the calvarium	^116^
	*MKKs*	*Mkk3*^*−/−*^*Mkk6*^*+/−*^ and *Mkk3*^*−/−*^	Similar phenotype to *Tak1*^*f/f*^;Osx-Cre mice	^116^
I-SMAD and ubiquitin-related regulation	*Smad6*	Col11α1 Prom-Smad6 Tg	Dwarfism and osteopenia; chondrocyte hypertrophy↓	^279^
		KO	Dwarfism; defects in axial and appendicular bones; delayed onset of hypertrophy	^280^
	*Smad6;Smurf1*	Col11α1::Tg	More severely delayed endochondral ossification than Smad6 Tg	^279^
	*Smad7*	ΔExon1	Osteopenia; BFR ↓ ; OC↑	^282^
		KO	Chondrocyte proliferation and hypertrophy ↓ ; shortened growth plate	^339^
		Prx1 Prom-Tg; Col11 Enh-Tg; Col11 Prom-Tg	Chondrodysplasia; mesenchymal condensation ↓ ; chondrocyte proliferation and hypertrophy↓	^283^
	*Smurf1*	KO	Bone mass ↑ ; BFR↑	^286^
		Col1α1 Prom-Tg	Osteopenia; BFR↓	^285^
	*Smurf2*	Col2α1 Prom-Tg	Osteoarthritis; intervertebral disc degeneration	^291,292^
		KO	Protection from age-related and DMM-induced osteoarthritis	^293^
		KO	Osteopenia ↓ ; OC↑	^296^
		KO	Enhanced BMP-induced ectopic bone formation	^294^
	*PLEKHO1*	Osterix-Cre	Protection from age-related bone loss	^289^
		Osterix Prom-Tg	Age-related bone loss	^289^
	*NEDD4*	Col1α1-Cre	Bone mass ↓ ; OB↓	^298^
		Col1α1 Prom-Tg	Bone mass ↑ ; OB↑	^298^
	*Jab1*	Osx-Cre	Dwarfism; trabecular bone mass↓	^303^
Antagonists	*Noggin*	Ocn Prom-Tg	Osteopenia; BFR↓	^250,253^
		Ocn-Cre	Osteopenia	^254^
		KO	Hyperplasia of cartilage; joint development failure; multiple skeletal defects related to neural tube and somite patterning (failure of neural tube closure, broad club-shaped limbs, loss of caudal vertebrae, a shortened body axis, and retention of a small vestigial tail)	^66,67^
	*Grem1*	Ocn-Cre	Bone mass ↑ ; BFR↑	^258^
		Ocn Prom-Tg	Bone fractures; bone mass ↓ ; BFR↓	^259^
	*FS*	Tg	Bone mass ↓ ; bone fractures	^262^
Co-receptors	*β-glycan*	KO	Defective palate development with OB↓	^267^
	*Nrps*	KO	Bone mass ↓ ; OB ↓ ; OC↓	^268^
	*Neogenin*	KO	Elongated growth plate; chondrocyte proliferation & apoptosis ↓ ; endochondral ossification↓	^276^
Other regulators	*Tmem53*	KO	Sclerosing bone	^308^
	*Endofin*	Col1 Prom-Endofin F872A Tg	Bone mass ↑ ; BFR↑	^304^

*Prom* promoter, *Enh* Enhancer, *Tg* transgenic, *KO* knockout, *CKO* conditional knockout, *BFR* bone formation rate, *OB* osteoblast, *OC* osteoclast, *OCY* osteocyte, *BMD* bone mineral density, *DN* dominant negative

Annotations.

(1) Col1α1 Prom-Tgfb1 H222D Tg, transgenic mice carrying a *Tgfb1 H222D* mutant under the control of *Col1α1* promoter; *Tgfb1 H222D* mutant is found in human Camurati-Engelmann disease (CED). This mouse model is also named as Tgfb1-CED or CED mice.

(2) Bp^J^ is the mouse model carrying *Gdf5* mutation (*bp-J* allele) which occurs spontaneously in the A/J strain. Bp is short for brachypodism.

**Table 2:** Human diseases related to TGF-β and BMP signaling in bone.

Gene	Disease	MIM#	Bone disorders	References
*NOGGIN, GDF5*	Symphalangism	185800, 186500, 184460, 615298	Ankylosis or synostosis of the interphalangeal joints	^59,60^
*NOGGIN*	Tarsal–carpal coalition syndrome	186570	Fusion of the carpals, tarsals, and phalanges; short first metacarpals causing brachydactyly; humeroradial fusion	^61^
*NOGGIN, BMP2, BMPR1B, GDF5*	Brachydactyly	611377, 112600, 113100	Brachydactyly	^60,62–64^
*TGFΒR1, TGFΒR2, TGFΒ2, TGFΒ3, SMAD2, SMAD3*	Loeys-Dietz syndrome	609192, 610168, 613795, 614816, 615582, 619656	Variable skeletal anomalies (including skeletal overgrowth, pectus deformity, osteoarthritis, hernias, etc.)	^72–76^
*ACVR1*	Fibrodysplasia ossificans progressiva	135100	Progressive heterotopic bone formation in muscles, tendons, ligaments, and joints	^122,123^
*TGFB1*	Camurati-Engelmann disease	131300	Osteosclerotic lesions in the long bones and skull with increased remodeling; osteoarthritis	^158^
*SMAD3, MAP2K1, LEMD3*	Melorheostosis	155950	Melorheostosis (special sclerosing bone disease)	^159–161,306,307^
*LEMD3*	Osteopoikilosis; Buschke-Ollendorff syndrome	166700	Sclerosing bone	^306,307^
*TMEM53*	Craniotubular dysplasia, Ikegawa type	619727	Hyperostosis; short stature in association with macrocephaly, dolichocephaly, or a prominent forehead	^308^
*FBN-1*	Marfan syndrome	154700	Variable skeletal anomalies including long bone overgrowth	^77^
*FBN-2*	Congenital contractural arachnodactyly	121050	Long limbs (dolichostenomelia) and long, slender fingers and toes (arachnodactyly), permanently bent joints (contractures)	^230^
*ADAMTSL2*	Geleophysic dysplasia	231050	Short stature, short extremities, and skeletal abnormalities	^243^
*ADAMTS10, ADAMTS17*	Weill-Marchesani syndrome	277600, 608328	Short stature brachydactyly, and ectopia lentis	^245^
*COL1A1, COL1A2*	Osteogenesis imperfecta	259420	A bone dysplasia characterized by bone deformities, fractures, and a high un-union rate caused by low bone mass and impaired bone quality	^235^
*EXT1, EXT2*	hereditary multiple exostoses	33700, 133701	Formation of cartilage-capped bony growths (osteochondroma) at the ends of the bones	^238^
*SKI*	Shprintzen-Goldberg syndrome	182212	A wide range of skeletal abnormalities including craniosynostosis, distinctive facial features, arachnodactyly, long limbs, pectus excavatum or carinatum, and scoliosis	^313^

## Overview of TGF-β and BMP Signaling Pathways

**Fig. 1:**
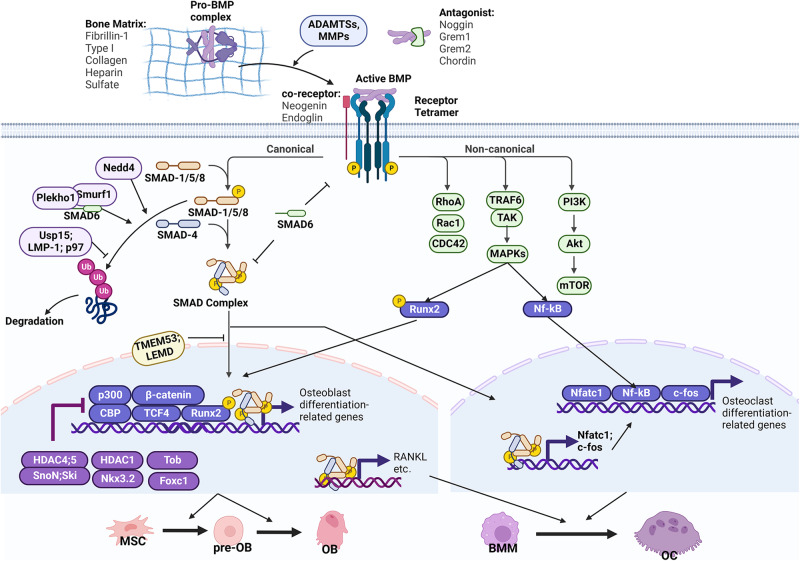
BMP signaling in bone remodeling. Pro-BMP proteins are bound with matrix proteins and are processed into active GF dimers through the proteolytic degradation of the PD by ADAMTSs and MMPs. BMP activity is regulated by bone matrix proteins in the extracellular region (FBN-1, COL1, HS) and by extracellular antagonists (Noggin, Grem1, Grem2, Chordin). Active BMPs bind to a receptor heterotetramer comprising of Type I and II receptors. Co-receptors such as Neogenin and endoglin might cooperatively bind to BMP receptors or ligands. The bindings ultimately result in the phosphorylation of type I receptors to transduce downstream signals through canonical and non-canonical pathways. In the canonical pathway, BMP-specific R-SMADs (SMAD-1/5/8) are activated by phosphorylation at C-terminal SSXS domains and form a complex with the Co-SMAD SMAD4 through the C-terminal MH2 domains. The activated SMAD complex then translocates into the nucleus to regulate the transcription of target genes. In the cytoplasm, I-SMAD SMAD6 inhibits the signaling by interfering with receptor–R-SMAD or SMAD complex formation. SMAD6 also cooperates with ubiquitin ligases (Smurf1 and Nedd4) to induce the ubiquitination and degradation of R-SMADs. Deubiquitinases such as Usp15 and LMP-1 positively regulate BMP signaling by antagonizing R-SMAD degradation. The nuclear translocation of the SMAD complex is regulated by nuclear envelope proteins such as TMEM53 and LEMD. In the osteoblast, the transcription function of the SMAD complex is regulated by co-transcription factors (p300, β-catenin, CBP, TCF4, Runx2) or repressors (HDAC4/5–SnoN; Ski complex, HDAC1–Nkx3.2 complex, Tob, Foxc1). In the non-canonical pathway, TRAF6 is recruited to the receptor to activate downstream factors, including MAPKs, PI3K, and small GTPases (Rho, Rac, Cdc42). MAPK signaling positively regulates activity of transcriptional factors, including Runx2 in osteoblasts and NF-кB in osteoclasts. Ultimately, BMP signaling promotes both osteoblast and osteoclast differentiation at all stages. OB osteoblast, pre-OB pre-osteoblast, BMM bone marrow monocyte, OC osteoclast.

**Fig. 2:**
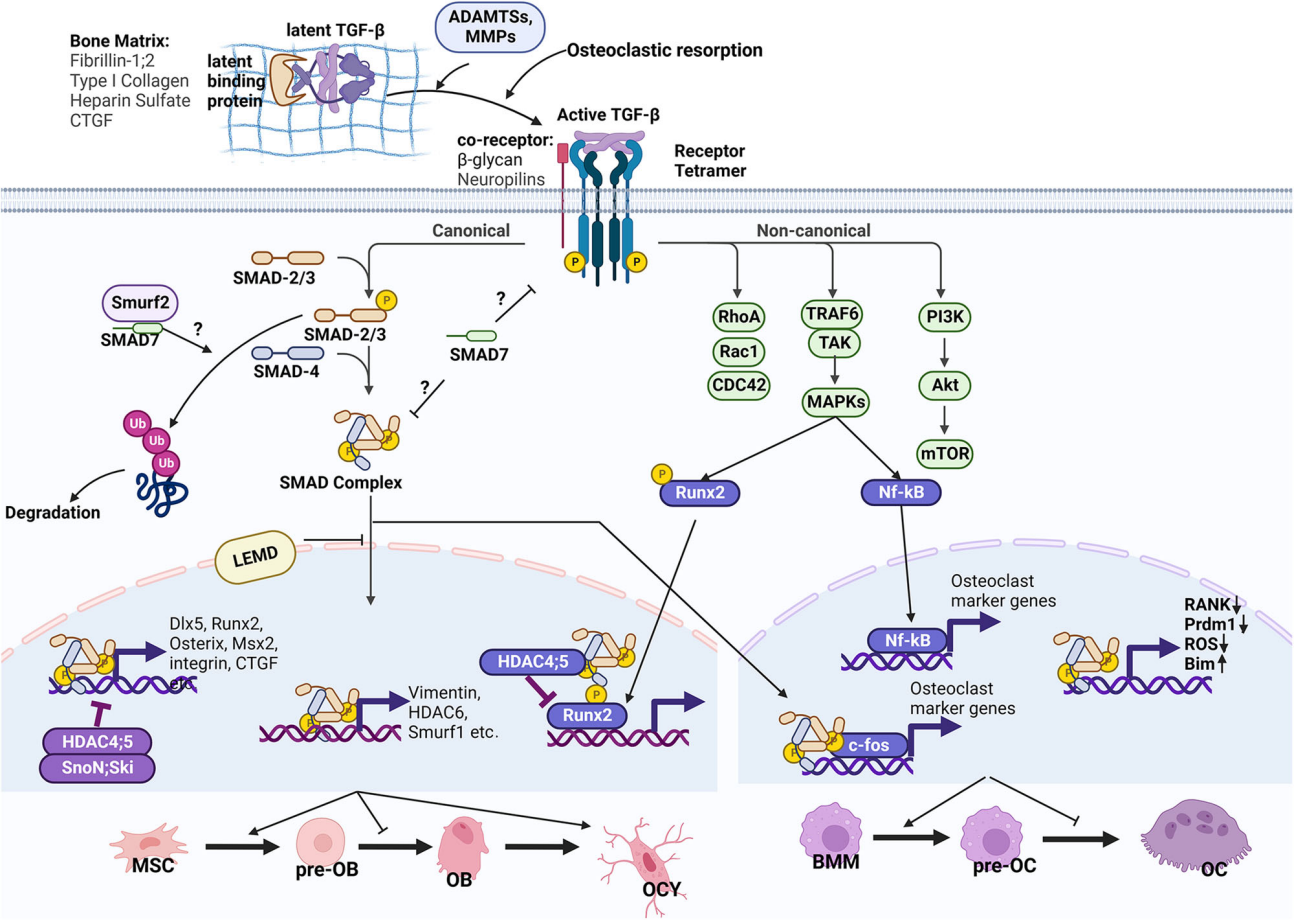
TGF-β signaling in bone remodeling. Besides bone matrix proteins, the latency of TGF-βs is also maintained by LTBPs, which bind TGF-β precursors to form the LLC. Active TGF-β peptides are released by osteoclastic bone resorption and proteolytic degradation by ADAMTSs and MMPs. Active TGF-β binds with a receptor heterotetramer, which transduces signals through canonical and non-canonical pathways like BMPs. Co-receptors β-glycan and Nrps facilitate ligand–receptor binding. In the canonical pathway, TGF-β-specific R-SMADs (SMAD-2/3) are activated by phosphorylation at C-terminal SSXS domains and form a complex with the Co-SMAD SMAD4 through the C-terminal MH2 domains. The activated SMAD complex would then translocate into the nucleus to regulate the transcription of target genes. I-SMAD SMAD7 and Smurf2 antagonize signaling activation in the cytoplasm. The nuclear translocation of the SMAD complex is regulated by nuclear envelope protein LEMD. In the osteoblast, the SMAD complex drives osteogenic gene expression (Dlx5, Runx2); however, it recruits HDAC4/5 to antagonize Runx2 activity and drives the expression of genes that inhibit osteoblast formation (HDAC6, Smurf1). The SMAD complex also plays dual roles in osteoclastogenic gene expression in the osteoclast. In the non-canonical pathway, TRAF6 is recruited to the receptor to activate downstream factors, including MAPKs, PI3K, and small GTPases (Rho, Rac, Cdc42). MAPK signaling positively regulates activity of transcriptional factors, including Runx2 in osteoblasts and NF-кB in osteoclasts. Ultimately, TGF-β promotes osteoblast and osteoclast early differentiation, limiting their later maturation. TGF-β also maintains the formation and property of osteocytes, while its mechanism remains unclear. OB osteoblast, pre-OB pre-osteoblast, OCY osteocyte, BMM bone marrow monocyte, OC osteoclast, pre-OC pre-osteoclast.

In the TGF-β and BMP signaling pathways, the dimeric ligands bind to heterotetrameric receptors comprising two type I and two type II receptors.^1–3^ This binding ultimately results in the phosphorylation and activation of a glycine-serine-rich domain within the type I receptor by the constitutively active type II receptor, transducing signals downstream through both suppressor of mothers against decapentaplegic homolog (SMAD)-dependent and -independent pathways (Figs. 1 and 2).^1–3^ The heterogenous ligand–receptor combinations and the dynamic regulations of TGF-β and BMP signaling result in versatile outcomes.

### Ligands and receptors: structure, diversity, and selectivity

More than 30 TGF-β superfamily members have been identified in mammals, including TGF-βs, BMPs/growth differentiation factors (GDFs), Nodals, and Activins. There are only a few studies addressing the roles of Nodals and Activins in the skeleton,^4–6^ which indicate that they play a negative role in osteogenesis. Activin A signaling was reported to increase in the skeleton of patients with chronic kidney disease-mineral bone disorder and might contribute to deranged bone turnover.^5^ In contrast, the functions of BMPs and TGF-βs in the skeleton have been more extensively investigated, and this review will mostly focus on them.

**Fig. 3:**
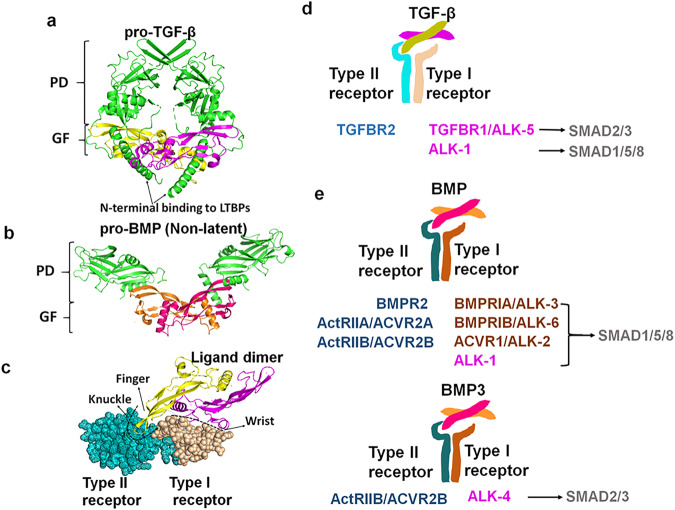
Structure and selectivity of TGF-β and BMP ligands and receptors. **a**–**c** Structures of pro-TGF-β1, pro-BMP9, and TGF-β1–TGFBR1–ALK5 complex were re-created from PDB files with accession codes 3RJR, 4YCI and 3KFD, respectively. Pro-TGF-β1 and pro-BMP9 both contain PD and GF dimer which non-covalently interact with each other. PD of pro-TGF-β1 interacts with LTBPs and conveys the latency of its GF. Unlike TGF-β1, PD of pro-BMP9 does not convey latency of its GF, and leave GF’s receptor-interacting domain ‘open’ (**a**, **b**). The active TGF-β1 is a GF dimer. Each monomer is like a “hand” with two β-strand “fingers” protruding from an α-helix “wrist”. The dimer binds the receptor complex at an interface composed of the “wrist” of one monomer and ‘fingers’ of the other monomer (**c**). **d,** **e** Ligands and receptors of TGF-β and BMP signaling in bone.

So far, more than 15 BMPs have been discovered in both humans and rodents. The skeletal system synthesizes many different BMPs, including BMP2, BMP3b/GDF10, BMP4, BMP5, BMP6, BMP7, BMP9/GDF2, BMP13/GDF6, and BMP14/GDF5.^7^ All three TGF-β ligands (TGF-β1, TGF-β2, and TGF-β3) are expressed in the skeleton.^8^ TGF-βs and BMPs are synthesized and secreted as pro-protein complexes which contain two N-terminal prodomains (PDs) non-covalently interacting with the C-terminal mature GF dimer (Fig. 3a, b).^7,8^ The PDs control the activity of GFs in different ways, including latency, localization, stability, and proper dimer formation.^9^ PDs of TGF-βs keep GFs latent in extracellular matrix (ECM) and control their bioavailability.^8,10^ Pro-TGF-β is also known as the small latent complex (SLC), with its PD known as latency-associated peptide (LAP). LAP interacts with a latent binding protein (LTBP) to form the large latent complex (LLC), which binds to ECM proteins such as fibrillin (FBN).^8,10^ The release and activation of TGF-βs from ECM involves dissociation at acidic pH or proteolysis by matrix metalloproteinases (MMPs) of osteoclasts.^11–13^ Our work showed that activation of TGF-β is abolished in ATP6i-deficient mice, whose osteoclasts were dysfunctional.^14^ In contrast, PDs of some BMPs do not convey their latency, including BMP4, BMP5, BMP7, and BMP9.^15–17^ Among them, BMP7 pro-protein is bound with FBNs in ECM to form proper signaling gradients,^18^ while BMP9 pro-protein is circulating,^15^ and the PD of BMP4 is also essential for the generation of active BMP4/7 heterodimer.^16^ Therefore, the activity of BMPs and TGF-βs are controlled by endopeptidases, and might also be controlled by matrix composites or matrix degradation enzymes if they are ECM-bound (discussed in more detail below).

Most TGF-β and BMP GF dimers are connected by a disulfide bond, although this is absent in a few BMPs (GDF3, GDF9, and BMP15).^19^ The disulfide-bonded dimeric structure is classically portrayed as a “hand”, in which two sets of anti-parallel β-strands form “finger extensions” that protrude from a central stabilizing “wrist” α-helix.^9^ They bind their receptors at a composite binding interface, which is formed by the “wrist” epitope of one monomer and the convex “knuckle” epitope of the “finger extensions” of the other monomer (Fig. 3c).^9^ Despite their structural similarity, TGF-β and BMP ligands possess different interfaces, also called hotspot regions or sites, to recognize diverse pairings of type I and type II receptor complexes (Fig. 3d, e). In the skeleton, TGF-βs usually bind to heterotetrameric receptors comprising of TGF-β type I receptor (TGFBR1)**/**Anaplastic lymphoma kinase 5 (ALK5) and TGF-β type II receptor (TGFBR2).^8^ Some studies also identified ALK1 as a second type I TGF-β receptor.^20,21^ The receptor-binding nature of BMPs is more heterogeneous than that of TGF-βs. In the skeleton exist three type II receptors for BMPs, including BMP type II receptor (BMPR2), Activin type IIA receptor (ActRIIA, ACVR2A), and Activin type IIB receptor (ActRIIB, ACVR2B). Moreover, there exist four type I receptors, including BMP type IA receptor (BMPRIA)/ALK3, BMP type IB receptor (BMPRIB)/ALK6, Activin type I receptor (ACVR1)/ALK2, and ALK1.^7,22–24^ Combinations of those type I and type II receptors form various heterotetrameric complexes, which possess different binding affinities for certain BMP ligands. For example, while both BMP7 and BMP14 bind to ALK6, only BMP7 binds to ALK2 and ALK3. Therefore, BMP7 and BMP14 play distinct but overlapping roles in skeletal development.^25^ Furthermore, BMP-2/4/9 stimulates bone formation preferably through ALK-1/3/6,^22,26,27^ while BMP3 antagonizes osteogenesis through binding to ActRIIB.^24,28^

### Canonical and non-canonical signaling

Upon binding to their receptors, the TGF-β superfamily transduces signals through canonical (Smad-dependent) and non-canonical (Smad-independent) signaling pathways (Figs. 1 and 2). In the canonical signaling pathways, eight SMAD proteins have been characterized in mammals (SMAD1–8), which could be classified into three subtypes: common partner SMAD (Co-SMAD, SMAD4), receptor-regulated SMADs (R-SMADs, SMAD-1, -2, -3, -5, and -8), and inhibitory SMADs (I-SMADs, SMAD-6 and -7). Binding of TGF-βs or BMPs with their receptors results in the phosphorylation and activation of R-SMADs via interaction with the C-terminal SSXS motif.^3,29^ The phosphorylated R-SMADs then form a complex with the Co-SMAD SMAD4 through their C-terminal MH2 domains, and translocate to the nucleus to regulate the transcription of target genes via binding to DNA through their N-terminal MH1 domains.^3,29^ In most cases, BMPs elicit the activation of R-SMADs SMAD-1, -5, and -8. In contrast, TGF-βs elicit the activation of R-SMADs SMAD-2 and -3 (Fig. 3d, e). Alternatively, TGF-βs also bind to ALK1 to transduce signals to SMAD-1, -5 and -8.^20,21^ Unlike R-SMADs and Co-SMAD, I-SMADs lack the DNA-binding MH1 domain and coordinate the negative regulation of canonical signaling, which is discussed in more detail in this review.

Alternatively, TGF-β or BMP receptors can transmit signals independent of SMAD proteins (Figs. 1 and 2).^3^ Upon ligand binding, TGF-β or BMP receptors associate with TNF receptor-associated factors (TRAFs) to promote their polyubiquitylation, which activates TGF-β activated kinase 1 (TAK1). TAK1 subsequently phosphorylates mitogen-activated protein kinases (MAPKs) or phosphoinositide 3-kinase (PI3K), which in turn phosphorylates and activates target transcription factors (i.e., nuclear factor kappa-B (NF-κB), runt-related transcription factor 2 (RUNX2)). TAK1 might also activate small G proteins, including Rac1 and Cdc42. Canonical and non-canonical signaling activation reciprocally regulates each other. On the one hand, the activation of non-canonical signaling could potentiate canonical signaling. For example, PI3K was shown to stabilize SMAD1 protein through GSK3 activation in vivo and in vitro, enhancing osteogenesis;^30^ furthermore, knockdown of extracellular signal-regulated kinase 1 (ERK1) was shown to inhibit TGF-β1-induced Smad3 phosphorylation in rat chondrocytes.^31^ On the other hand, non-canonical signaling could also antagonize SMAD activity. For example, MAPK might phosphorylate Smad1 to recruit Smurf1 for its cytoplasm retention and degradation.^32^ NF-κB could interact with Smad4 and antagonize its transcriptional activity to suppress BMP2-induced bone formation.^33^ ERK signaling is reported to increase the expression of Smurf1 to inhibit BMP’s function in osteoblasts.^34^ An uneven activation of TAK1 over SMADs by c-Abl directs the expression of p16(INK4a) to control mesenchymal stem cell (MSC) maintenance and inhibit osteoblast differentiation.^35^

### Target transcriptome

The Smad complex recognizes consensus DNA sequences, namely Smad-binding element (SBE) or BMP-responsive element (BRE), to regulate gene expression. The SBE element, also known as the GTCT motif or its complementary extended CAGAC sequence, has been previously identified.^36^ Smad1 and Smad5 were shown to also recognize GC-rich motifs (GGCGC), termed BRE, in certain BMP-responsive genes.^36^ As such, some target genes have been identified for TGF-β and BMP signaling, including *Id-1*, *Gremlin*, *noggin*, *follistatin (FS)*, *Smad6*, and *BambI*.^37^ However, the target transcriptome of TGF-β and BMP signaling also varies greatly among cell types and pathological conditions, due to variable cofactors and chromatin structure and accessibility. Therefore, the development of chromatin immunoprecipitation followed by sequencing (ChIP-seq), formaldehyde-assisted isolation of regulatory elements followed by sequencing (FAIRE-seq) or CUT&Tag-seq, as well as ATAC-seq and RNA-seq techniques enables the genome-wide analysis of SMAD-binding and SMAD-responsive sites in a cell type-specific manner. Omata et al. ^38^ performed ChIP-seq combined with FAIRE-seq in osteoclasts to analyze the TGF-β-responsive and receptor activator of nuclear factor-κB ligand (RANKL)-regulated genes. Their results indicated the cooperation of Smad2/3 with c-Fos during osteoclastogenesis.^38^ Yu et al. ^39^ used RNA-seq, ATAC-seq combined with H3K27Ac CUT&Tag-seq, to analyze deregulated transcription factor networks in Bmp2-deficient osteoblasts, revealing that over 80% of deregulated elements are directly targeted by transcription factors such as RUNX2, DLX5 (Distal-Less Homeobox 5), MEF2C (MADS box transcription enhancer factor 2), OASIS (old astrocyte specifically induced substance), and KLF4 (Krüppel-like factor 4). These transcriptional factors may function together with or downstream of SMAD proteins to regulate the biological outcomes induced by BMP2. With RNA-seq and ChIP-seq techniques, Yan et al. ^40^ identified that Smad4 directly binds to the regulatory region of the *Runx2* promoter, which contributes to osteoblast differentiation and chondrocyte hypertrophy. The diverse target transcriptome could be explained by the notion that SMAD proteins recruit different transcription co-regulators on the chromosomes, which is discussed in more detail in this review.

## TGF-β and BMP signaling in skeleton development

The mammalian skeleton is formed through intramembranous ossification (i.e., calvarial bones) or endochondral ossification (i.e., appendicular bones and axis bones).^41,42^ During intramembranous ossification, condensed mesenchymal cells are directly differentiated into osteoblasts and osteocytes.^41,42^ During endochondral ossification, condensed mesenchyme undergoes chondrogenesis to form cartilage primordium, which develops into a cartilage anlage of embryonic bone shape, surrounded by the perichondrium.^41,42^ The cartilage anlage further develops into growth plates at the two epiphyseal ends, which are layered with chondrocytes in continuous differentiation stages (resting, proliferative, pre-hypertrophic, and hypertrophic).^41,42^ The hypertrophic chondrocytes undergo terminal differentiation and apoptosis and are gradually replaced by bone structures in the metaphyseal part. Multiple signaling pathways (i.e., Hedgehog, fibroblast growth factor (FGF), parathyroid hormone-related protein (PTHrP), BMP, and TGF-β) cooperate to determine the morphology of the skeleton in the cartilage primordium stage and modulate bone growth and maturation in the growth plates.^41,42^ Our work showed that transcriptional factor complexes Runx1/Cbfβ and Runx2/Cbfβ control chondrocyte proliferation and hypertrophy during growth plate development.^43–47^ Here, we will discuss the specific roles of BMP and TGF-β signaling in skeleton development, especially endochondral ossification.

### BMP signaling in skeleton development

**Fig. 4:**
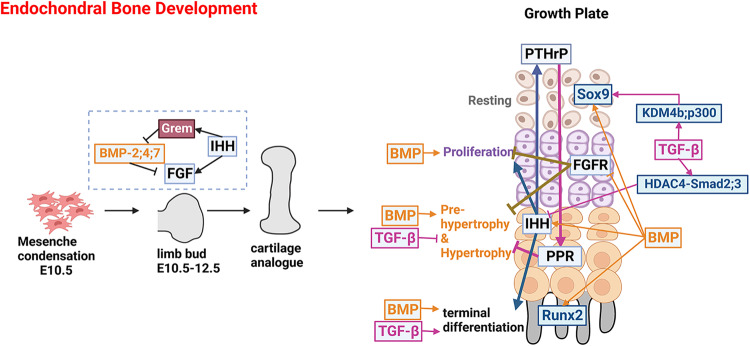
TGF-β and BMP signaling in endochondral bone development. Endochondral bone development begins with the condensation of mesenchyme, which develops into limb bud, cartilage analogue, and embryonic bone with a well-organized growth plate in a step-wise fashion. In the early stage, BMPs are expressed in the anterior and posterior margins of the limb bud. IHH induces the expression of BMP antagonist Gremlin in the posterior margins. Gremlin prevents BMPs from downregulating FGF production which feeds back to maintain IHH production. The BMP-IHH-FGF regulatory loop establishes the dorsal-ventral and anterior-posterior axes of the limb bud and determines limb patterning. In the growth plate, BMP signaling promotes chondrocyte proliferation and differentiation at all stages, while TGF-β promotes the terminal differentiation of chondrocytes while inhibiting hypertrophic differentiation. BMP positively regulates IHH signaling to promote chondrocyte proliferation through the IHH-PTHrP loop, negatively regulates FGF signaling, a negative regulator of chondrocyte proliferation and hypertrophy, and promotes Runx2 activity to enhance hypertrophic and terminal differentiation. In contrast, TGF-β decreases IHH expression. BMP and TGF-β promote Sox9 expression or activity, favoring cartilage matrix production.

BMP signaling plays critical roles in multiple stages of skeletogenesis, including MSC condensation, cartilage primordium formation, skeleton patterning, and growth plate development (Fig. 4). As mentioned earlier, BMP signaling consists of a variety of ligands and receptors with heterogeneous binding affinities and patterns, which produce variable physiological outcomes. BMP ligands have different expression patterns during skeleton development, delineating their diverse physiological functions. For example, Bmp14 and its receptor Alk6 have a restricted expression pattern in appendicular bones.^25^ Consistently, mice carrying the *Bmp14* mutation, *Alk6* null mutation, or both display malformation of appendicular bones but not axis bones.^25,48,49^ Bmp-2, -4, -7, and -14 (GDF5) are expressed in the early stage of skeletal development, indicating their roles in the initiation of skeletogenesis.^25,50–52^ Consistently, embryonic deletions of *Bmp-2*, *-4*, *-7*, or *-14* genes result in malformed skeletons.^25,50–52^ Among them, MSC-specific *Bmp-2* and *-4* double knockout (DKO) mice displayed the most severe malformation, highlighting their critical functions during embryonic skeletal development.^51^ At the molecular level, loss of BMP impairs prechondrogenic differentiation at mesenchyme condensations due to expressional loss of key chondrogenic transcription factors, including Sox-5, -6, and -9.^48^

Moreover, BMP signaling is critical in early limb bud development (Fig. 4). BMP-2, -4, and -7 are expressed in both the anterior and posterior margins of limb bud mesenchyme.^51^ BMP antagonist Gremlin is also expressed in the posterior margins of the limb bud.^53^ Msx2-Cre-mediated *Bmp4, Bmp2*, and *Bmp7* deletion in apical ectodermal ridge (AER) cells resulted in the disruption of dorsal-ventral polarization of mesenchyme and AER disorganization.^54–57^ During distal progression of limb bud development, Sonic Hedgehog (SHH) activity leads to the upregulation of the BMP antagonist Gremlin in the posterior mesenchyme (or zone of polarizing activity) to prevent BMPs from downregulating FGF production in the AER, which feeds back to maintain SHH production.^58^ Mutations of genes in BMP signaling, including *NOGGIN*, *GDF5*, *BMP2*, and *BMPR1b*, are associated with human diseases characterized by symphalangism or brachydactyly^59–64^ (Table 2). Manipulating the aforementioned genes in mice emulates these human disease phenotypes while also having additional autopod patterning defects, such as polydactyly and missing phalange elements.^25,48–52,65–67^

BMP signaling promotes chondrocyte proliferation and differentiation at all stages of growth plate development (Fig. 4). In the growth plate, expression is found for Bmp2, Bmp4, and Bmp5 in the perichondrium, Bmp2 in hypertrophic chondrocytes, and Bmp7 in proliferating chondrocytes.^7^ Severe chondrodysplasia and shortened long bones are observed in chondrocyte-specific *Bmp2* conditional knockout (CKO) and *Bmp-2* and -*4* DKO mice but not in *Bmp4* CKO mice, indicating that Bmp2 outweighs Bmp4 in regulating chondrocyte differentiation during growth plate formation.^50^ As for their receptors, ALK-2, -3, and -6 play redundant roles during skeleton development since all the chondrocyte-specific DKO mice display a more severe, perinatal, lethal chondrodysplasia phenotype than the single gene CKO mice, exhibited by delayed chondrocyte proliferation, matrix production, hypertrophic differentiation, and terminal differentiation.^25,48,49,65^ Similar phenotypes could also be observed in mice with R-Smad or Co-Smad proteins deleted specifically in chondrocytes.^40,50,68,69^ Conversely, augmentation of BMP signaling accelerates chondrocyte maturation and cartilage expansion, as observed in chick limbs loaded with constitutive active (CA) forms of BMP receptors and mouse models with activated BMP signaling.^66,67,70^ BMP regulates chondrocyte proliferation and hypertrophic differentiation through several different mechanisms. First, BMPs maintain the expression of Sox9, a master chondrogenic transcription factor.^48,71^ Second, BMP signaling induces the expression of Indian Hedgehog (Ihh),^27,65,69^ a cytokine critical for maintaining proliferating chondrocyte pool. Third, BMPs negatively regulate FGF signaling by inhibiting the expression of FGFR1.^65^ FGF signaling was shown to inhibit chondrocyte proliferation and hypertrophy through STAT and MAPK signaling.^65^ Furthermore, BMP/Smad4 promotes the expression and activity of Runx2, which positively regulates chondrocyte hypertrophy and ossification.^40^

### TGF-β signaling in skeleton development

Like BMPs, TGF-β signaling is also indispensable for skeleton development. In humans, deregulated TGF-β signaling caused by the mutations of TGFΒR2, TGFΒ2, TGFΒ3, SMAD2, SMAD3, and FBN-1 is associated with Loeys-Dietz syndrome or Marfan syndrome, both of which are characterized by various skeletal anomalies such as long bone overgrowth^72–77^ (Table 2). In mice, deletion of *Tgfbr2* abolished TGF-β signaling and resulted in severe defects in calvarial, appendicular, and axis bones.^78^ TGF-β also plays a critical role in joint morphogenesis. Tgfbr2 deficiency results in ankylosis of the interphalangeal joints and missing or incomplete intervertebral discs (IVDs).^78–81^ TGF-βs regulate the expression of several joint morphogenic genes, including Noggin, Wnt9a, GDF5, and MCP-5 (monocyte chemotactic protein-5).^81,82^

Unlike BMPs, the roles of TGF-β in chondrogenesis are differentiation stage-dependent. At an early stage of differentiation, TGF-β signaling is not required to initiate chondrogenesis but limits chondrogenesis for osteoblast lineage commitment. Neither MSC-specific nor chondrocyte-specific *Tgfbr2* CKO mice experience difficulty in forming the primordium.^78–80^ Deletion of *Alk5* in mice also led to a thinner perichondrium accompanied by ectopic cartilaginous tissues protruding into the perichondrium.^83^ At a later stage of differentiation, TGF-β signaling prevents chondrocyte hypertrophy while promoting terminal differentiation.^78,81,84^ TGFBR2 is the only type II receptor for TGF-βs, and deletion of *Tgfbr2* effectively abolished TGF-β signaling and resulted in severe defects in calvarial, appendicular, and axis bones.^78^ However, severe skeleton defects were not observed in TGF-β1, 2, and 3 single gene KO mice,^85–88^ indicating that they play redundant roles. During terminal differentiation, Tgfbr2 deficiency accelerated the transition from pre-hypertrophic to hypertrophic chondrocyte while delaying ossification.^78,81,84^ Similar defects were observed in the chondrocyte-specific *Smad*-*2* and -*3* CKO and DKO mice, while Smad2-deficient mice displayed a more severe phenotype, indicating that Smad2 plays a more critical role than Smad3 in endochondral bone development.^89^ Smad2 is shown to inhibit the expression of Ihh at the transcriptional level to a greater extent than Smad3.^89^

## TGF-β and BMP signaling in bone formation and remodeling

Throughout the life of humans, bone tissues undergo continuous remodeling, with bone resorption carried out by osteoclasts and bone formation by osteoblasts.^90,91^ The differentiation program from skeletal MSCs to osteoblasts is regulated by multiple signaling pathways (i.e., IGF, WNT, Hedgehog, parathyroid hormone (PTH), TGF-β, and BMP) and transcription factors (i.e., Runx2, Dlx5, Osterix, β-catenin).^91^ Our works showed that Runx1/Cbfβ and Runx2/Cbfβ control osteoblast differentiation and lineage commitment.^44,92–94^ Osteoclasts differentiate from bone marrow monocytes/macrophages, a process driven by two cytokines: M-CSF and RANKL.^90^ Osteoclast differentiation is also controlled by key transcription factors like c-Fos, NF-кB, and nuclear factor-activated T-cells 1 (NFATc1).^90^ Osteocytes are terminally differentiated osteoblasts embedded in the mineralized matrix.^95^ Osteocytes localized in the lacuna of bones have multiple dendritic extensions to connect with nearby osteocytes and cells on the bone surface, forming a specialized structure called the lacuna-canalicular network.^95^ Osteocytes directly participate in perilacuna bone remodeling and modulate osteoclast and osteoblast functions through paracrine pathways.^95^ An imbalance between osteoclast and osteoblast activity and dysregulated osteocyte function will disturb bone homeostasis, resulting in bone metabolic diseases like osteopenia and osteosclerosis. Here, we summarize the role of TGF-β and BMP signaling in regulating osteoclast, osteoblast, and osteocyte formation and function. Multiple genetic mutations in TGF-β and BMP signaling are associated with various human sclerosing symptoms (Table 2). Genome-wide studies and single-gene analysis also identified genetic polymorphisms of several genes in both pathways associated with bone mass, including TGF-β1, BMP2, BMP4, SMAD9, SMAD2, Noggin, SOSTDC1, GREM2, NAB1, and SPON1.^96–106^ The involvement of TGF-β and BMP signaling in postnatal bone homeostasis is also substantiated by extensive in vivo, in vitro and ex vivo studies.

### BMP signaling in bone formation and osteoblast differentiation

BMPs were first discovered and mainly referred to as osteogenic proteins (Fig. 1). BMP2 is considered the gold standard for bone regeneration and has been clinically applied to promote fracture healing and spinal fusion.^107,108^ Additionally, BMP-2, -4, -6, -7, and -9 are also osteogenic in vitro and in vivo.^107–110^ However, endogenous BMP2 might have a unique and indispensable function in fracture healing since *BMP2* CKO mice also have frequent fractures that fail to heal, which is not observed in *BMP4* CKO mice.^111,112^ BMP9 has been recently found to be resistant to endogenous antagonists such as Noggin and BMP3b, providing a candidate alternative to BMP2 for treating fracture healing.^109,110^ In addition, mouse models were generated with BMP canonical and non-canonical signaling suppressed in osteoblasts, including *Alk2* CKO mice,^113^ *BmprII* dominant-negative transgenic mice,^114^ *ActRIIB*-null mice,^115^ *Smad1* CKO mice,^50^ *Tak1* CKO mice,^116^ *p38* CKO mice,^117^ and Smad4-deficient mice.^118,119^ All aforementioned mice exhibited osteopenia phenotypes, further substantiating the osteogenic role of BMP signaling in promoting osteoblast differentiation and matrix production.

Moreover, hyperactivated BMP signaling leads to heterotopic ossifications (HO). One of the major side effects of BMP implementation in bone healing is inducing HO in muscle tissues.^120^ Musculoskeletal trauma-induced HO in muscles and tendons at a high ratio is associated with hyperactivated BMP signaling.^121^ Antagonizing BMP signaling activation is proposed to be a potential treatment preventing trauma-induced HO.^121^ Fibrodysplasia ossificans progressiva (FOP; MIM #135100), a genetic disorder manifesting progressive HO, is caused by gain-of-function mutations (R260H and G356D) of *ALK2/ACVR1*, the type I receptor of BMPs (Table 2).^122,123^ In animal and cell models of FOP, the ACVR1 mutants transduce hyperactivated Smad1/5/8-dependent signals downstream of either BMP or ALK2, explaining the pathomechanism of FOP (Table 1).^122,124–130^ In contrast, under normal circumstances, BMP-ACVR1 activates Smad1/5/8 and Activin A-ACVR1 activates Smad2/3 signaling.^131^ Retinoic acid receptor γ (RARγ) agonist Palovarotene, which suppresses BMP signaling,^132^ has been recently approved by the U.S. Food and Drug Administration for FOP treatment based on its Phase III trial.^133^ Besides Palovarotene, selective ALK2 inhibitors (BLU-782, Phase I; INCB00928, Phase II; Saracatinib) and the Activin A neutralizing antibody also showed potential to alleviate FOP symptons.^128,134,135^

At the molecular level, BMPs promote osteogenesis through several different mechanisms. Firstly, BMP signaling positively regulates the activity of Runx2, an osteoblast master transcription factor. Smad1 physically interacts with Runx2 to bind to OSE2 sites on its target gene.^136^ Runx2 is also phosphorylated by BMP non-canonical signaling (TAK1-MEK-p38 or ERK), promoting its association with the coactivator CREB-binding protein (CBP).^116^ BMP also stabilizes Runx2 through promoting its acetylation by p300.^137,138^ Secondly, frequent crosstalk between BMP and WNT signalings promotes the osteogenic program. For example, transcription factor 4 (TCF4)/β-catenin complex physically interacts with the SMAD complex on the corresponding DNA-binding sites^139^; ablation of Smad4 causes cleavage of β-catenin and depletion of the WNT receptor, a low-density lipoprotein receptor (Lrp5)^118^; expression of LGR4, an orphan receptor and WNT regulator, is also induced by BMP2.^140^ Thirdly, BMP signaling induces the expression of several osteogenesis-related transcription factors, including Msx2, Runx2, Dlx5, KLF10, Forkhead box C1 (Foxc1), Foxc2, and Dlx3.^139,141–143^ Fourthly, BMP2 also induces the expression of PLCβ1 (phospholipase C β1) and IHH, both of which promote osteoblast differentiation.^144,145^ Additionally, SMAD1 dislodges Hoxc-8 from its DNA-binding sites to induce osteoblastic gene expression.^146^ Moreover, BMP signaling positively regulates mTORC1 activity to promote osteoblast activity.^147^ Our work showed that Runx1 regulates osteoblast differentiation through promoting BMP signaling, by controlling *Bmp7* and *Alk3* expression at transcriptional level.^94^

However, BMP signaling also has adverse effects on bone formation. BMP limits the proliferation of preosteoblasts and antagonizes osteogenesis in osteoblast progenitors.^147^ BMP signaling might also negatively regulate mineralization and collagen maturation.^148,149^ At the molecular level, Alk3 induces the expression of WNT antagonists, DKK1 (Dickkopf-related protein 1), and sclerostin (SOST).^150^ BMP2 promotes an interaction between Smad1 and Dvl-1 (Drosophila dishevelled gene) that restricts β-catenin activation.^151^ Smad4 also competitively interacts with Tcf and Lef (lymphoid enhancer binding factor) proteins to inhibit the transcriptional activity of β-catenin.^119^ Collectively, BMP antagonizes bone formation through perhaps inhibiting WNT/β-catenin signaling.

### TGF-β signaling in bone formation and osteoblast differentiation

As discussed above, BMP signaling limits osteoprogenitor proliferation while promoting osteogenesis afterward. In contrast, TGF-β signaling promotes osteoprogenitor proliferation and osteogenesis at the early stage of differentiation while inhibiting bone formation at the later stage (Fig. 2). Many mouse models with impaired TGF-β signaling have been generated, including *Tgfb1*-null mice, MSC-specific and osteoprogenitor-specific *Tgfbr2* CKO mice, *Alk5*-null mice, and *Smad3*-null mice.^83,152–157^ Those TGF-β signaling-dificient mice displayed significant bone loss with reduced osteoblast number, suggesting that TGF-β is anabolic for bone formation.

Conversely, hyperactivated TGF-β signaling increased bone mass. In humans, gain-of-function mutations in TGFB1 are associated with Camurati-Engelmann disease (CED; MIM #131300), characterized by osteosclerotic lesions within the long bones and the skull.^158^ Mice carrying the same *tgfb1* mutation mirror the phenotype seen in humans.^152^ Somatic SMAD3-activating mutations in humans are associated with endosteal pattern melorheostosis (Leri’s disease; MIM #155950), characterized by asymmetric exuberant bone formation.^159,160^ Interestingly, osteogenesis of SMAD3-activating mutant cells is stimulated by TGF-β while inhibited by BMP2,^159,160^ indicating that SMAD3 links the reciprocal regulation between BMP and TGF-β. Furthermore, activating mutations of mitogen-activated protein kinase kinase 1 (*MAP2K1*) in non-canonical TGF-β signaling also caused sporadic melorheostosis.^161^ At the molecular level, TGF-β positively regulates the expression of Runx2, Osterix, Dlx5, and Msx2 to initiate the osteogenic program.^153^ TGF-β1 induces the expression of integrin Vα5 to promote osteoblast adhesion.^162^ TGF-β1-SMAD signaling also regulates the expression of connective tissue growth factor (CTGF), a matrix protein that positively regulates osteoblast differentiation and function.^163^

During the late stage of osteoblast differentiation, TGF-β signaling inhibits bone formation. TGF-β, SMAD3, and SMAD2 are shown to inhibit osteogenesis in vitro.^164–167^ Smad3 interacts with Runx2 and recruits histone deacetylase 4 (HDAC4) and 5 (HDAC5).^166^ HDAC4 and HDAC5 deacetylate Runx2 to facilitate its degradation.^137^ TGF-β regulates the expression of various signaling proteins involved in osteoblast formation. TGF-β induces the expression of vimentin, which negatively regulates the activity of ATF4, an osteogenesis-related transcription factor.^168^ TGF-β induces the expression of HDAC6, which distorts primary cilia to impair mechanical-stimulated osteogenesis.^169^ TGF-β induces the expression of Smurf1, which antagonizes osteogenic signaling such as BMP.^34,170^ TGF-β also inhibits the expression of IGF-1, a bone anabolic cytokine.^171^ In vivo, CKO of *Tgfbr2* in mature osteoblasts results in high bone mass in mice.^172^ Qiu et al.^172^ revealed that Tgfbr2 forms a complex with PTHrP for endocytosis. With the deletion of *Tgfbr2*, PTH signaling is hyperactivated to produce excessive bone mass.^172^ PTH signaling also reciprocally regulates TGF-β signaling by inducing LTBP-1, TGF-β1, and Smad3 expression.^173,174^

### BMP and TGF-β signaling in osteoclast differentiation

BMP signaling promotes osteoclast differentiation both directly and indirectly. BMP promotes osteoblast-induced osteoclast formation through upregulating the RANKL/osteoprotegerin (OPG) ratio (Fig. 1). Disruption of Alk3, Alk2, or Smad4 in osteoblasts or osteocytes results in an unexpected increase of bone mass in mice due to the decreased RANKL/OPG ratio causing less osteoclast formation.^150,175–178^ Alk2 and Alk3 signaling upregulate WNT antagonists (i.e., Sost) to inhibit WNT activation, and the latter regulates osteoclast formation by inhibiting the RANKL/OPG ratio.^150,175,176^ Therefore, BMP might be essential to promote osteoblast-osteoclast coupling in bones requiring extremely active remodeling, such as during regeneration.^145^

BMP signaling also stimulates osteoclast formation directly (Fig. 1). BMPs (i.e., BMP2, BMP7) stimulate and BMP inhibitor dorsomorphin blocks osteoclast formation and bone resorption.^179–182^ Consistently, deletion of *ALK2*, *ALK3*, *SMAD1/5*, or *SMAD4* also impairs osteoclastogenesis.^180,182,183^ At the molecular level, BMP signaling promotes the expression or activity of osteoclastic transcription factors. BMPRII couples with RANK to activate p-Smad1/5/8 and NF-κB signaling simultaneously.^181^ Smad1/5/8 promotes the nuclear translocation of NFATc1.^182^ Moreover, Smad1/5 induces the expression of c-Fos and Nfatc1.^180^

Unlike BMPs, TGF-β regulates osteoclast formation in a dose- and stage-dependent manner (Fig. 2). Low-dose TGF-β induces, whereas high-dose TGF-β inhibits, migration of osteoclast precursors to the bone resorption pits.^184^ TGF-β at the monocyte stage promotes, while at the later differentiation stage antagonizes, osteoclast formation. TGF-β regulates multiple signalings in regulating osteoclast differentiation. TGF-β-induced p38 activation and Smad2/3 cooperation with c-Fos as a co-transcription factor favor osteoclast differentiation.^38,185^ TGF-β inhibits RANK expression, blocks Prdm1 activity to induce Irf8 and Bcl6 expression, and upregulates ROS production to block MAPK signaling to antagonize osteoclast differentiation.^185–187^ TGF-β also upregulates Bim expression to induce osteoclast apoptosis.^188,189^

### The role of TGF-β signaling in osteocytes

While the well-known osteocyte marker SOST is an antagonist of WNT and BMP signaling and a critical regulator of skeletal homeostasis, knowledge about the role of BMP signaling in osteocytes is very limited. In contrast, recent studies brought up the physiological role of TGF-β in regulating osteocyte formation and function. TGF-β-Smad3 signaling has been previously shown to inhibit the transition of osteoblasts into osteocytes.^156^ In mature osteocytes, TGF-β signaling was recently demonstrated to play a critical role in maintaining its perilacunar-canalicular network and function (Fig. 2). In mice, intrinsic osteocytic TGF-β signaling promotes the perilacunar-canalicular remodeling of the osteocyte to control bone quality.^190,191^ Specific loss of TGF-β signaling in the osteocyte reduces osteocyte connectivity, impairing fluid dynamics and osteocyte exposure to mechanical stimulation.^192^ Conversely, administration of TGF-β1 increases osteocyte connectivity in bone tissue and an MLO-Y4 cell line by inducing connexin43 and pannexin1 expression.^193^ TGF-β3 was also shown to maintain the osteocyte differentiation of MLO-Y4 cells in an osteoblast-osteocyte co-culture 3D system as determined by stable E11 and osteocalcin mRNA expression.^194^ Furthermore, intrinsic osteocytic TGF-β signaling is also essential for the mechanosensing property of articular cartilage. Mice with impaired TGF-β signaling in osteocytes have thicker subchondral bone plates, high SOST levels, and more severe cartilage degeneration in an injury-induced osteoarthritis (OA) model.^195^

## TGF-β and BMP signaling in articular cartilage homeostasis

Joints are organized structures allowing constrained motion. They are formed by adjacent bones with articular cartilage covering the bone surface and contain the synovial lining of the joint cavity. Articular chondrocytes govern articular cartilage homeostasis via their ability to modulate ECM production and degradation, whose imbalance causes degenerative joint diseases such as OA. In the diseased joint, chondrocytes undergo abnormal hypertrophic and terminal differentiation, followed by tearing of the cartilage matrix, focal calcification, and ectopic bone (osteophyte) formation.

**Fig. 5:**
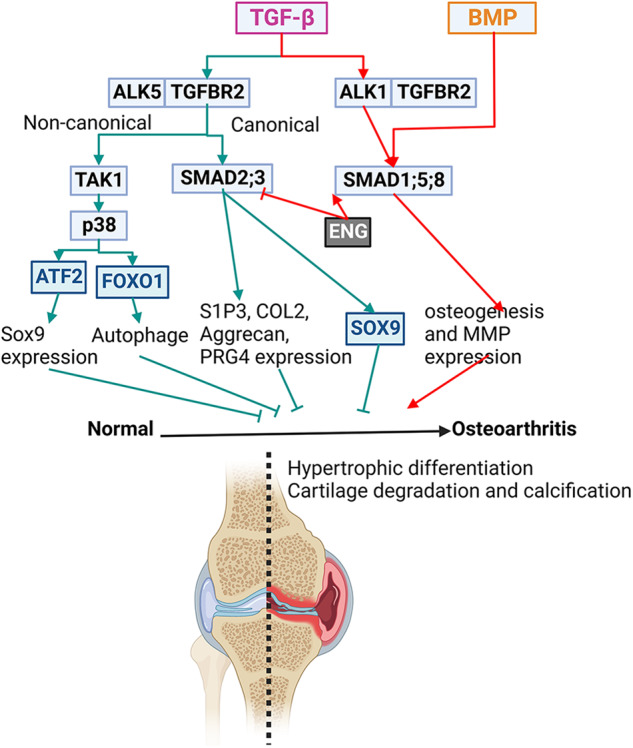
TGF-β and BMP signaling in cartilage homeostasis. Postnatal cartilage homeostasis is maintained by matrix production and degradation balance, and the imbalance results in cartilage tearing and joint diseases like osteoarthritis. TGF-β plays dual roles in cartilage homeostasis. To protect cartilage health, TGF-β, through binding ALK5, activates SMAD-2 and -3 and TAK1-p38 signaling, which enhances the Sox9 expression and activity and promotes autophagy activity and matrix protein production. Conversely, TGF-β, through binding ALK1, activates SMAD-1, -5, and -8, like BMPs, which promotes MMP production and osteogenesis to aggravate cartilage degeneration.

On the one hand, TGF-β signaling plays a critical role in maintaining articular homeostasis (Fig. 5). TGF-β1-coupled biomaterials have been proposed as a therapeutic method for cartilage repair.^196,197^ TGF-β signalings protect articular cartilage by inhibiting chondrocyte hypertrophy and apoptosis,^198,199^ promoting cartilage matrix synthesis,^200–202^ and antagonizing inflammatory cytokine production.^203,204^ In humans with grade 3 OA, genetically modified allogeneic human chondrocytes that express TGF-β1 show significant improvement in knee joint function and reduce pain severity.^205^ Animal models with inhibited canonical and non-canonical TGF-β signaling are prone to developing OA, including dominant-negative *Tgbr2* transgenic mice,^206^ mice with postnatal cartilage-specific deletion of *Alk5*, *Tgfbr2*, or *Tak1*,^71,207–209^ *Smad3*-null mice,^199^ and dominant-negative *p38* transgenic mice.^210^ Pharmacological inhibition of TGF-β signaling also leads to an OA-like phenotype in rodents.^71,211,212^ At the molecular level, the reduction of TGF-β canonical signaling induces the death of articular chondrocytes.^198^ TGF-β non-canonical signaling induces the phosphorylation and activation of ATF2 and FoxO, which inhibits OA by upregulating the expression of Sox9 and autophagy proteins.^71,213^ Inhibition of TGF-β activity enhances BMP and S1P (sphingosine 1-phosphate) signaling, which accelerates chondrocyte maturation and matrix degradation.^199,214^ Abolished TGF-β activity also alters IGF and FGF signaling and upregulates the expression of biosynthesis-related genes and electron transport chain-related genes, contributing to chondrocyte hypertrophy.^215^ Our work showed that Runx1 protects cartilage homeostasis through promoting TGF-β signaling.^216^

On the other hand, TGF-βs also promote the progression of OA. TGF-β expression is increased in osteoarthritic cartilage and joints with ankylosing spondylitis.^217–220^ Furthermore, mechanical loading during OA could induce TGF-β1 secretion.^221^ Excessive TGF-β signaling is detrimental to joint degeneration. Notably, CED patients or mouse models carrying gain-of-function mutations of *TGFB1* are prone to developing OA.^221,222^ Suppression of TGF-β signaling by deleting *Tgfbr2* in nestin-positive MSCs ameliorates the development of OA after anterior cruciate ligament transection (ACLT) compared to a control.^221^ The contradictory roles of TGF-β in OA have been linked to the opposite regulatory functions of its type I receptors, ALK1 and ALK5, to transduce signals to SMAD1/5/8 and SMAD2/3, respectively, in chondrocytes.^20,21^ ALK1 signaling is destructive by inducing the expression of matrix-degrading enzyme MMP-13. In contrast, ALK5 signaling is protective by inducing the expression of matrix proteins aggrecan, type II collagen, and PRG4 (proteoglycan 4).^207,223^ In addition, ALK1 opposes TGF-β-ALK5-induced phosphorylation of SMAD3 and inhibits the expression of chondrogenic genes induced by TGF-β, including fibronectin and type II collagen.^20^ Furthermore, ALK1/ALK5 ratio is increased in aging and osteoarthritic cartilage in mice.^223^ Disturbed balance between ALK1 and ALK5 signalings might contribute to articular cartilage degeneration.^223^ In addition, TGF-β signaling also promotes the clustering of nestin-positive MSCs, leading to the formation of marrow osteoid islets accompanied by high levels of angiogenesis to deteriorate OA condition.^221^

Abnormal BMP activation is associated with OA since BMP accelerates chondrocyte terminal differentiation.^199^ Recently, Occhetta et al. ^224^ found that selective inhibition of BMP signaling helps control differentiation of MSCs into chondrocytes at precisely the stage as those in articular cartilage. As cultured chondrocytes usually undergo terminal differentiation, this finding indicates that targeting BMP signaling provides a strategy for cartilage regeneration. BMP activity also needs to be inhibited spatially in vivo during development or in postnatal cartilage to prevent further chondrocyte differentiation as well as the overexpression of its antagonists, such as Gremlin.^224^

## Regulation of TGF-β and BMP signaling in bone

TGF-β and BMP signaling is regulated at multiple levels from extracellular space to nucleus (Figs. 1 and 2). Extracellularly, matrix proteins such as FBNs and collagens control the latency of TGF-βs and BMPs; metalloproteinases contribute to the release and activation of TGF-β and BMP peptides; antagonists interrupt the binding of TGF-β and BMP ligands to their receptors. At the cell membranes, co-receptors such as β-glycan and endoglin (ENG) facilitate the ligand–receptor interactions. In the cytoplasm, I-SMAD, ubiquitin ligases, and deubiquitinases regulate the activation and stability of SMAD complexes. Nuclear envelope proteins control the transport of SMAD complexes from cytoplasm to nucleus. Various transcription co-factors and epigenetic factors cooperate with SMAD complexes in the nucleus to regulate their transcription activity. Here, we will summarize how those regulators coordinate BMP and TGF-β signaling in bone and cartilage.

### Latency and release control of the ligands

LTBPs interact with LAPs and active TGF-β peptides to form the LLC. LTBP is indispensable for the latency, correct folding, and secretion of TGF-β. It is also essential for storing TGF-β in the ECM through interactions with platform proteins. Currently, four different LTBPs (LTBP-1–4) have been identified.^225^ Among them, LTBP-3 is the most studied. *Ltbp-3*-null mice have impaired TGF-β signaling, exhibiting multiple skeletal malformations and an OA-like phenotype.^226,227^ Impaired TGF-β signaling in *LTBP-3* null cells also reduced proliferation and osteogenic potential.^228^

The FBN microfibril network controls the latency of TGF-βs and BMPs by serving as their reservoir in the bone and cartilage matrix. The major component of the microfibril network, Fbn, binds the LLCs or CPLXs through its unique N-terminal region. Fbn-1 and -2 are both found to be expressed in the cancellous bone.^229^ In humans, mutations in *FBN-1* and *FBN-2* cause pleiotropic manifestations in Marfan syndrome (MIM #154700) and congenital contractural arachnodactyly (MIM #121050), respectively^77,230^ (Table 2). *Fbn-1*-null mice had systemic sclerosis due to abnormal activation of both TGF-β and BMP signalings.^231^ However, Fbn-2 deficiency in mice induced a low bone mass phenotype due to improper activation of TGF-β inhibiting osterix expression and increasing osteoblast-induced osteoclast formation.^231,232^ Microfibril-associated glycoprotein-1 (MAGP1) is another constitutive component in microfibril network.^233,234^ *Magp1*-null mice, resembling *Fbn-2*-null mice, developed progressive osteopenia due to abnormal activation of TGF-β.^233,234^

Type I collagens (COL1s), COL1A1 and COL1A2, also serve as reservoirs for TGF-βs in the bone matrix. Autosomal dominant mutations of *COL1* in humans cause osteogenesis imperfecta (OI; MIM #259420), a bone dysplasia characterized by bone deformities, low bone mass, poor bone quality, frequent fractures, and high non-union rate (Table 2).^235^ Cartilage-associated protein (CRTAP) catalyzes the maturation of COL1 by 3-hydroxylation, and its mutations also cause OI. Both *Col1a2*^*G610c/+*^ and *Crtap*^*–/–*^ mouse models recapitulated OI phenotypes due to excessive TGF-β signaling.^236,237^ Importantly, anti-TGF-β antibody 1D11 treatment both corrects the bone phenotype and improves fracture healing in the OI mouse model, highlighting the potential of targeting TGF-β signaling in treatment for OI.^236,237^

Heparin sulfate (HS) is abundant in the cartilage matrix and binds to latent TGF-βs and BMPs. EXT1 and EXT2 are Golgi-resident glycosyltransferases participating in the biosynthesis of HS.^238^ Mutations of *EXT1* and *EXT2* in humans cause hereditary multiple exostoses (MIM #133700, #133701), a human autosomal skeletal disorder characterized by the formation of cartilage-capped bony growths (osteochondroma) at the ends of the bones, due to excessive BMP signaling.^238^ Mouse models with CKO of *Ext1* in cartilage tissue develop osteochondroma and enhanced chondrocyte hypertrophy due to increased BMP-SMAD activity.^239–241^

CTGF is a cartilage matrix protein bound to latent TGF-βs.^242^ The Ctgf-deficient mice developed more severe OA than control mice due to increased TGF-β-SMAD activity.^242^

A disintegrin and metalloproteinase with thrombospondin motifs (ADAMTS) and MMPs help release active TGF-βs and BMPs from ECM via a proteolytic process. As reported, ADAMTSL2, ADAMTS17, and ADAMTS10 regulate skeletal development by activating TGF-βs or BMPs. Mutations of *ADAMTSL2* in humans are associated with recessive geleophysic dysplasia (MIM #231050), characterized by short stature, short extremities, and skeletal abnormalities^243^ (Table 2). Delhon et al. ^244^ generated whole-body and chondrocyte-specific Adamtsl2-deficient mice, both of which displayed skeletal abnormalities reminiscent of the human phenotype due to impaired TGF-β signaling. Mutations of *ADAMTS10* and *ADAMTS17* in humans cause Weill-Marchesani syndrome (WMS; MIM# 277600, 608328) and WMS-like syndromes, characterized by short stature and brachydactyly^245^ (Table 2). *Adamts17*^*–/–*^ mice recapitulated WMS phenotype with shortened growth plate due to impaired BMP activation.^246^ TGF-βs or BMPs are also activated by proteolytic processing of the PD by MMPs, including MMP-2, -9, and -13.^11–13^

### Extracellular antagonists

Noggin is a twelve-membered cystine knot protein and a critical antagonist of BMP ligands in bone. The crystal structure of the BMP–Noggin binding complex has been previously determined, showing that Noggin acts by sequestering the ligand in an inactive state.^247^ Noggin has a similar expression pattern to BMPs in bone during prenatal and postnatal development.^248,249^ In animal and cell models, Noggin blocks osteoblast formation by inhibiting BMP activation. Administration of Noggin suppresses osteogenesis,^250,251^ and neutralizing Noggin promotes osteoblast differentiation.^252^ Mice with conditional overexpression of Noggin showed dramatic decreases in bone mineral density and bone formation rates.^250,253^ However, deletion of *Noggin* in mature osteoblasts resulted in more osteoclast formation and osteopenia.^254^ Whether the detrimental impact on bone is attributed to an excessive presence of BMP or whether Noggin plays a BMP-independent role in skeletal homeostasis remains uncertain. In humans, mutations of *NOGGIN* are associated with various ankylosis deformities, including proximal symphalangism (SYM1: MIM #185800), multiple synostosis syndrome (SYNS: MIM#186500), tarsal–carpal coalition syndrome (TCC; MIM#186570), and stapes ankylosis with broad thumb and toes (SABTT; MIM#184460) (Table 2).^59^ Noggin mutant cells from ankylosis patients outperformed healthy cohorts in osteogenic differentiation capacity due to enhanced BMP activity.^255^ Noggin prevents cranial suture closure by inhibiting BMP signaling during cranial bone development. Therefore, Noggin downregulation also contributes to syndromic craniosynostoses.^256^

Gremlin-1 and Gremlin-2 (Grem1 and Grem2) are DAN family proteins and extracellular antagonists of BMPs. The structure of the Grem2–GDF5 complex reveals that two Grem dimers bind perpendicularly to each ligand monomer as a stable aggregate-like structure, which is not observed in Noggin and FS.^257^ Suppression of Grem promotes osteogenesis in vivo and in vitro due to sensitization of BMP signaling.^258^ Consistently, the osteoblast-specific *Grem1* CKO mice are osteosclerotic, and osteoblast-specific *Grem1*-overexpressing mice are osteopenic.^258,259^ Grem1 expression also defines a population of skeletal stem cells in the bone marrow required for both bone remodeling and fracture repair, as reported by Worthley et al. ^260^ Grem1^+^ stem cells can self-renew and differentiate into osteoblasts, chondrocytes, and reticular marrow stromal cells while lacking the capacity to develop into adipocytes.^260^

FS binds and neutralizes several different members in the TGF-β superfamily, including BMPs, Activin A, GDF11, and myostatin/GDF8. Among them, BMP promotes osteogenesis, myostatin and Activin A are negative regulators of bone mass, and the role of GDF11 in bone homeostasis is controversial.^6,261,262^ So far, FS was mostly reported to play anti-osteogenic roles.^262,263^ FS restricts BMP2 action in osteoblastogenesis in vitro, and mice overexpressing FS exhibited spontaneous bone fractures.^262,263^

Chordin is another well-established BMP antagonist and has a role in early embryonic neural development. Very few studies characterized the role of chordin in bone, showing that the expression of chordin is inversely related to osteoblast and chondrocyte differentiation.^264,265^

### Co-receptors

β-glycan, also regarded as the TGF-β type III receptor, acts as a membrane-anchored proteoglycan to enhance TGF-β association with the TGFBR2–TGFBR1 complex, but its soluble form may also associate with TGF-βs, activins, or BMPs to inhibit signal transduction. β-glycan is expressed in osteoblasts and promotes osteogenesis in vivo and in vitro.^266,267^ *β-glycan*-knockout embryos displayed reduced vascular and osteoblast differentiation.^267^

Neuropilins (Nrps) interact with TGFBR1 to promote downstream signaling. Nrp2 is expressed in both osteoblasts and osteoclasts, and *Nrp2*-knockout mice had increased osteoclast number, decreased osteoblast number, and low bone mass.^268^ While Nrps also bind and transduce signals downstream of semaphorins, how much its role in bone homeostasis is attributed to TGF-β signaling is still unclear.

ENG may bind to BMP ligands or BMPR2 receptors to facilitate signal transduction while associating with TGF-β1 or TGF-β3 for signaling through ALK3. ENG enhances BMP2-induced osteogenesis of periodontal ligament (PDL) cells in osteoblasts.^269^ ENG also acts as a co-receptor for BMP9 and BMP10 to induce osteogenesis in conjunction with ALK1.^15,270^ ENG is also expressed in human chondrocytes, and its expression increases in the chondrocytes of OA patients.^271,272^ Yet its function in chondrocytes remains controversial.^271–273^ ENG enhances Smad1/5 signaling and inhibits Smad2/3 activation to promote cartilage matrix protein production.^271^ However, knockdown of ENG also impaired cartilaginous tissue formation.^274^

Neogenin binds to BMP receptors.^275^ *Neogenin*-null mice have impaired limb development and endochondral ossification due to decreased BMP-SMAD signaling and Runx2 expression.^276^

### Regulation machinery in the cytoplasm

#### I-SMADs Smad6 and Smad7

In the cytoplasm, the signaling is mainly negatively regulated by I-Smads (Smad6 and Smad7). I-SMADs inhibit the receptor-mediated activation of R-Smads through several mechanisms including interfering with type I receptor–R-Smad interaction, recruiting ubiquitin ligases to induce type I receptor or R-SMAD protein degradation, and interfering with the formation of R-SMAD–Co-SMAD complex.^277^ Therefore, the inhibitory functions of I-SMADs largely depend on their direct interactions with the type I receptors or R-SMADs. I-SMADs bind R-SMADs or receptors through their C-terminal MH2 domains, which show high similarity between SMAD6 and SMAD7. However, their N-terminal Leu-rich motifs (LRMs) have a low similarity rate of 36.7%, laying down the structural basis for their functional differences. SMAD6 prefers to inhibit BMP signaling, whereas SMAD7 inhibits TGF-β and BMP signaling.^277^ Like Noggin, *SMAD6* mutations in humans also cause craniosynostosis due to the augmentation of BMP signaling.^278^ Smad6 transgene blocked BMP activation and led to osteopenia and dwarfism in mice.^279^ *Smad6*-null mice exhibited axial and appendicular skeletal development defects, with an expanded hypertrophic zone attributed to increased BMP responsiveness.^280^ Smad6 also recruits Smurf1 to ubiquitinate and degrade Runx2 to inhibit osteoblast differentiation.^281^ In contrast, SMAD7 might be anabolic for bone. Partial loss of *Smad7* decreased bone formation and increased bone resorption.^282^ Smad7 overexpression impacts both early and late stages of chondrocyte differentiation due to downregulation of both BMP and TGF-β signalings.^283^

#### E3 ubiquitin ligases

I-SMAD recruits ubiquitin ligases to degrade target proteins, mainly the neural precursor cell expressed developmentally downregulated 4 (NEDD4) subfamily of HECT (homologous to the E6-accessory protein) E3 ubiquitin ligases, such as Smurf1, Smurf2, and Nedd4.

Smurf1, together with Smad6, catalyzes the poly-ubiquitination and degradation of multiple targets with an osteogenic function, such as SMAD-1, -5 and -8, MEKK2, and Runx2.^281,284–287^ Therefore, Smurf1 has an anti-osteogenic function. Double overexpression of Smad6 and Smurf1 delayed ossification more severely than Smad6 overexpression alone.^279^ The *Smurf1* transgenic mice also had significantly reduced bone formation, while *Smurf1*-null mice had increased bone mass.^279,285,286^ A chalcone derivative inhibiting Smurf1 activity promotes local spinal fusion and systematic bone formation in mice, indicating that targeting Smurf1 is a potential treatment for bone healing.^288^

Pleckstrin homology domain-containing family O member 1 (PLEKHO1) associates with Smurf1 to promote the ubiquitination of Smad1/5 to inhibit BMP signaling and bone formation.^289^ Furthermore, the expression of PLEKHO1 increased during aging, indicating its involvement in aging-related bone loss.^289^

Smurf2 is a negative regulator of BMP and TGF-β signaling. Smurf2 is detrimental to cartilage homeostasis by antagonizing TGF-β signaling. Smurf2 overexpression promotes chondrocyte maturation, causing spontaneous OA and accelerated age-related IVD degeneration.^290–292^ Smurf2 deficiency protects both young and aged mice from surgically-induced OA.^293^ Smurf2 negatively regulates BMP signaling to inhibit osteogenesis.^294^ Smurf2 was proposed to induce degradation of the TGF-β receptors, Smad2, and Smad3. However, neither of those proteins increased in *Smurf2*-null mice.^295^ Instead, mono-ubiquitination of SMAD3 was reduced to favor SMAD complex formation in the absence of Smurf2, which mediates the interaction between SMAD3 and vitamin D receptor to modulate RANKL production and osteoclast formation.^295,296^

Nedd4 regulates the degradation of Smad1 to antagonize BMP signaling and inhibit bone formation.^297,298^ *Nedd4* overexpression in osteoblasts increases bone mass, and *Nedd4* deletion in osteoblasts reduces bone formation.^298^

#### Deubiquitination

Deubiquitylating enzyme USP15 stabilizes ALK3 to enhance BMP activation and osteoblast differentiation.^299^ USP15 also inhibits OA progression by deubiquitinating ERK2 and enhancing ERK2-induced TGF-β/SMAD2 signaling.^300^

Osteogenic LIM mineralization protein (LMP-1) antagonizes SMAD ubiquitination to promote TGF-β and BMP activation. LMP-1 interacts with Smurf1 to prevent Smad-1 and -5 ubiquitination, and interacts with Jab1 to prevent Smad7-induced Smad-4 and -5 ubiquitination.^301^

Valosin-containing protein (VCP)/p97, together with its adaptor nuclear protein localization 4 (NPL4), interacts explicitly with Smurf1 and delivers the ubiquitinated Smurf1 for degradation. Mutation of *VCP/p97* causes rare forms of Paget’s disease of bone (PDB)-like syndromes by increasing BMP activity.^302^

COP9 signalosome is a protein complex with isopeptidase activity responsible for the deneddylation of RING ubiquitin ligases (CRL) by catalyzing the hydrolysis of NEDD protein CRL. Jab1, also known as Csn5/Cops5, is a crucial subunit of the COP9 signalosome. *Jab1* deletion in preosteoblast reduced the response to TGF-β and BMP signaling, impairing osteoblast differentiation and reducing the trabecular bone number.^303^

#### Phosphatases and kinases

TGF-β and BMP receptor activity is also regulated by phosphorylation and dephosphorylation. Endosome-associated FYVE-domain protein (endofin), previously implicated in regulating membrane trafficking, also recruits protein phosphatase 1 catalytic subunit (PP1c) to exert a negative regulative effect on BMP signaling by dephosphorylating the BMP type I receptor.^304^ A single point mutation of endofin (F872A) disrupts endofin–PP1c interaction and sensitizes BMP signaling to increase osteogenesis in vitro and in vivo.^304^ Casein kinase II (CK2) phosphorylates the ALK3 receptor to block its activity, reducing BMP2’s osteogenic effects on osteoblasts in patients with osteoporosis.^305^

### Regulation in the nucleus

#### Nuclear envelope proteins

Transport of the SMAD complex into the nucleus is controlled by the nuclear pore complex (NPC), comprising multiple copies of ~30 different proteins located on the nuclear envelope. As the boundary between the cell nucleus and cytoplasm, the nuclear envelope comprises a double-membrane sheet, the inner nuclear membrane (INM) and the outer nuclear membrane (ONM). LEM domain containing 3 (LEMD3), an INM protein and transmembrane protein 53 (TMEM53) have been reported to regulate bone BMP and TGF-β signaling. Loss of function of LEMD3 results in unique sclerosing bone disease spectrums, osteopoikilosis (MIM #166700), melorheostosis (MIM #155950) and Buschke-Ollendorff syndrome (BOS; MIM #166700) (Table 2).^306,307^ LEMD3 has been shown to antagonize BMP and TGF-β by interacting with SMAD-1, -2, -5, and -9. TMEM53 inhibits BMP signaling in osteoblast lineage cells by blocking cytoplasm-nucleus translocation of SMAD1/5/8 specifically.^308^ In humans, TMEM53 was identified as a susceptibility gene for osteoporosis in several studies,^309,310^ and was recently associated with a previously unknown type of sclerosing bone disease (Table 2).^308^

#### Transcription repressors

Ski is a nuclear proto-oncogene protein homolog of the avian sarcoma viral (v-ski) oncogene and is a repressor of TGF-β and BMP signaling by inhibiting the transcription activity of SMAD complex.^311^ It also recruits histone deacetylases HDAC4 and HDAC5 as co-repressors.^312^ *SKI* mutations in humans cause Shprintzen-Goldberg syndrome (GOSHS; MIM #182212),^313^ which share multiple skeletal anomalies with Marfan syndrome caused by mutations of *FBN-1* (Table 2). Both diseases are associated with enhanced TGF-β and BMP signaling.

SnoN, a Ski proto-oncogene homolog, also interacts with the SMAD complex. A negative feedback mechanism, regulated by SnoN, can be evoked by TGF-β to oppose BMP signaling in chondrocytes and osteoblasts.^314,315^ SnoN and Ski might have different functions since they are differently recruited by Smad2 and Smad3.^89^

Nkx3.2 is a transcriptional repressor expressed in the sclerotome and developing cartilage, where it activates the chondrocyte differentiation program via a BMP-dependent manner. Mechanistically, Nkx3.2 forms a complex with histone deacetylase 1 (HDAC1) and Smad-1 and -4 in a BMP-dependent manner through its homeodomain and NK domain to repress gene expression cooperatively.^316^

Tob is a member of the emerging family of anti-proliferative proteins and negatively regulates BMP signaling in osteoblasts by directly interacting with Smad-1, -5, and -8 in the nucleus. *Tob*-null mice have a greater bone mass due to an increased number of osteoblasts.^317^

FOXC1 could repress the transcriptional activity of SMAD-1 and -5 to modulate the expression of BMP-responsive genes to prevent osteoblast differentiation.^318^

#### Transcription co-factors

Runx2 is a critical transcription factor in promoting osteoblast differentiation and chondrocyte hypertrophy. Runx2 is physically and functionally associated with Smad proteins in osteoblasts and chondrocytes.^319,320^ Javed et al. ^320^ reported that BMP-induced osteogenesis is blunted in *Runx2*-null cells, and Runx2 with mutations in Smad-interacting domain (HTY (426–428)) is only marginally functional in promoting osteoblast differentiation at early stages.

TCF4 and β-catenin are the transcription factors activated by canonical WNT signaling and are anabolic for osteogenesis. They form a complex with Smad proteins on the promoter of osteoblastic genes and recruit co-activators such as CBP or p300, cooperatively regulating the expression of early osteoblast genes such as Dlx5, Msx2, Runx2, and osterix.^139^

Sox9 is the key chondrogenic transcription factor. Sox9 interacts with Smad2/3 on the Col2 enhancer region in a TGF-β-dependent manner and recruits co-activators such as CBP or p300 to promote transcription.^321^

c-Fos, a key osteoclastic transcription factor, interacts directly with SMAD-2 and -3 to promote osteoclast diffrentiation.^38^

Lysine demethylase 4B (KDM4B), a histone demethylase whose expression is induced by TGF-β, potentiates TGF-β-mediated chondrogenesis of human MSCs in a positive feedback loop.^322^ Mechanistically, KDM4B removes the silencing H3K9me3 marks on the *SOX9* promoter to facilitate SMAD3 binding and transcription.^322^

## Conclusion and Perspectives

BMP and TGF-β signaling is essential in embryonic skeleton development and postnatal bone and cartilage homeostasis. Dysregulated TGF-β and BMP signaling causes numerous hereditary skeletal diseases in humans. For example, excessive TGF-β signaling in humans due to *TGFB1*, *SMAD3*, or *MAP2K1* gene mutations leads to a spectrum of sclerosis symptoms. Excessive TGF-β activation is also associated with OI. *NOGGIN*, *SMAD6*, or *ALK2* mutations augment BMP signaling to cause craniosynostosis or HO. Mutations of *FBN-1/2* or *SKI* enhance both TGF-β and BMP signalings to cause similar skeletal anomalies in humans. Mutations of *ADAMTS* block TGF-β and BMP activation and lead to short stature anomalies. Moreover, genome-wide association studies have identified several genes in TGF-β and BMP signaling associated with bone density. Most phenotypes were recapitulated in genetic mouse models carrying those disease-associated mutations, which provide disease models for pathomechanism studies and drug screening. Targeting TGF-β and BMP signaling effectively cures their associated skeletal disorders in diseased mouse models and clinical trials, such as OI and HO.

TGF-βs and BMPs belong to the same family, share structural similarities, and transduce signals through both SMAD-dependent and -independent pathways. However, they recruit different receptors to activate independent sets of SMAD proteins, laying down the molecular basis for their diverse functions. For example, BMPs, but not TGF-βs, are essential for limb bud outgrowth. In chondrocytes, BMPs promote differentiation at all stages; in contrast, TGF-β promotes chondrocyte early development but antagonizes its hypertrophy. BMPs promote osteoblast and osteoclast differentiation and are applied to improve fracture healing. Meanwhile, TGF-β signaling plays dual roles in osteoblast and osteoclast formation. Moreover, BMP and TGF-β also play opposite roles in articular cartilage homeostasis.

Genetics and molecular biology studies have advanced our understanding of the function and dynamic regulations of BMP and TGF-β signaling in the skeleton. However, more precise knowledge is still in demand and might promote the development of effective therapeutic strategies to treat related skeletal disorders. Future directions may lie in answering the following questions:

1. Why do BMP and TGF-β signalings have dynamic functions? As reviewed here, this question could be partially answered by the diversity of ligand–receptor combinations and the complex intracellular regulatory network that causes the dynamic readout of the TGF-β and BMP signaling. In particular, the BMP signaling pathway comprises multiple ligands and receptors that interact promiscuously with one another. A series of works from Dr. Michael B. Elowitz’s group demonstrated that the promiscuous ligand–receptor interaction systems of BMP signaling are critical for its dynamic regulations.^323–325^ Their work elucidated how the BMP pathway processes multi-ligand inputs using a repertoire of computational mechanisms, including ratiometric sensing, balance detection, and imbalance detection. Since cells have different expression patterns of receptors and ligands, the promiscuous interaction system allows a small number of ligands, acting in combinations, to address the issue of a larger number of individual cell types.
2. What transcriptional mechanism operates to bring about the diversity of transcriptional outcomes that arise in different cell types in response to the same ligand? Answering this question would require using state-of-the-art techniques such as ChIP-seq, co-IP/MS, ATAC-seq, and CUT&Tag-seq. DNA and histone modification status varies in different cell types and might alter the affinity of SMAD complex binding with the chromosomes. Therefore, analyzing the epigenetic marks on the transcription factor binding sequences would help answer this question. Characterization of the receptor–ligand interaction mode and chromatin status in specific cell contexts might also explain why TGF-β signaling has stage-dependent functions in most skeletal cells.
3. How to circumvent the side effects of BMPs and TGF-βs when applying them in clinical settings? Excessive BMP and TGF-β signaling is associated with multiple anomalies in bone tissues. Thus, further study and intervention are needed to prevent those side effects when applying BMPs and TGF-βs in clinical settings. Although TGF-β signaling maintains cartilage degeneration, hyperactivated TGF-β signaling aggravates OA. Despite its dual functions, TGF-β signaling is still proposed as a potential treatment to alleviate OA, although it needs more study to design the proper timing and dose for the treatment.
4. How to safely and effectively modulate BMP and TGF-β signaling in skeletal disorders caused by their dysfunctions? Targeting BMP and TGF-β signaling is proposed as the therapeutic strategy to treat OI, HO, or osteosclerosis disorders while effective treatment is still under development.
